# Case report: Study of a bulky melanoma mimicking sarcoma^☆^

**DOI:** 10.1016/j.ijscr.2025.110923

**Published:** 2025-01-23

**Authors:** Emma Quack, Hicham Fatouaki, Emma Afri, Romina Mastronicola, Gilles Dolivet

**Affiliations:** a Department of Head and Neck Surgery, Institut de Cancérologie de Lorraine, 6 avenue de Bourgogne, 54519 Vandœuvre-lès-Nancy, France; b CRAN, CNRS, UMR 7039, Université de Lorraine, Vandœuvre-lès-Nancy, France; c Faculté d'odontologie de Lorraine, Université de Lorraine, Vandœuvre-lès-Nancy, France

**Keywords:** Case report, Bulky melanoma, Mimicking, Dermis, Sarcoma

## Abstract

**Introduction:**

Large melanomas, while relatively uncommon, present significant diagnostic challenges due to their size and potential to mimic other malignancies, leading to delays in appropriate treatment. Initial misdiagnosis is a substantial concern, impacting patient outcomes. This case highlights the importance of immunohistochemistry in cancer diagnosis, and of appropriate therapeutic management, which here included excision surgery of the tumor mass.

**Case presentation:**

This case report details a 75-year-old male who presented with a large mass on his left arm, initially hypothesized as a liposarcoma. Advanced imaging (MRI) and immunohistochemical analysis revealed the mass to be a superficial spreading melanoma expressing SOX10, PS100, Melan-A, HMB-45, and PRAME.

**Clinical discussion:**

The patient later presented other skin lesions. As melanoma increase the risk of developing skin tumors, it is conceivable that the lesions may be interconnected. However, the lack of invasion beyond the dermis and the absence of metastasis suggest otherwise. The patient's psychological profile was investigated as another potential risk factor of cancer development. Inflammatory microenvironment was also observed, linked to the bacterial superinfection in the site of the initial tumor.

**Conclusion:**

This case underscores the considerable diagnostic challenges caused by large melanomas, especially their potential for mimicking other malignancies. Biopsy, incorporating advanced imaging and immunohistochemistry, is crucial for accurate and timely diagnosis, enabling appropriate management and improving patient outcomes. This case emphasizes that, when possible, surgery should be performed regardless of the size of the tumor. Long-term surveillance is vital given the increased risk of subsequent skin cancers in such patients.

## Highlights

• A 75-year-old male's arm mass, thought to be a liposarcoma, was correctly diagnosed as a bulky melanoma using immunohistochemistry
• When possible, surgery should be performed regardless of the size of the tumor, as it allowed favorable patient outcome.
• Psychological stress and bacterial infection can influence tumor growth, as seen in this patient.

## 1 Introduction

Melanomas represent a form of skin cancer resulting from an abnormal proliferation of melanocytes and their incidence has been increasing since the 1980's [1]. They account for 10 % of skin cancers. There are 4 main types of skin melanoma: superficial extensive, Dubreuilh, nodular and acrolentiginous melanoma. Characteristic features of melanoma include asymmetry, irregular borders, color variation, a diameter greater than 6 mm, and evolving features (the “ABCDEs”). While sarcomas may sometimes mimic melanoma, the reverse is less frequently reported.

This case report illustrates a large, atypical melanoma initially thought to be a liposarcoma, emphasizing the need for thorough investigation, proper excision surgery and differential diagnosis in such cases.

The patient was managed at Institut de cancérologie de Lorraine.

## 2 Case report

**Fig. 1:**
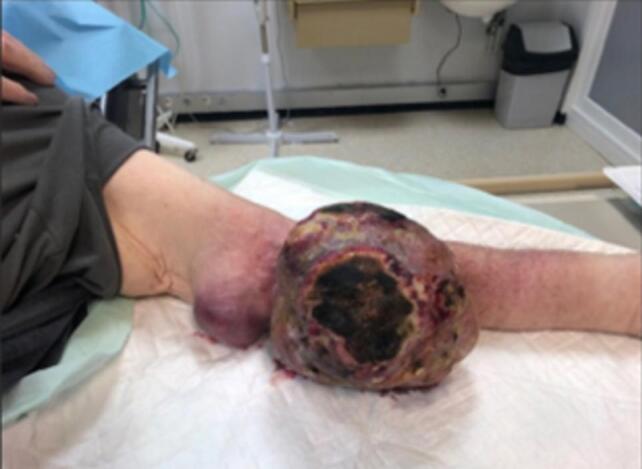
Appearance of the left forearm.

A 75-year-old male presented to the emergency department in November 2023 with a large (approximately 30 cm diameter), oval mass on his left upper arm, evolving for at least one year. The larger mass (16 × 13 cm) extended from the elbow to the mid-forearm; a smaller mass (6.4 × 4.5 cm) was located opposite the lower third of the humerus (Fig. 1).

The aspect of the mass indicated the potential presence of a liposarcoma, necessitating management by a specialized medical facility. Consequently, the patient was referred to Institut de Cancérologie de Lorraine (ICL) for appropriate treatment. During the initial consultation with the specialist, the suspicion of sarcoma persisted, prompting a biopsy in the excision area in accordance with sarcomas management guidelines.

**Fig. 2:**
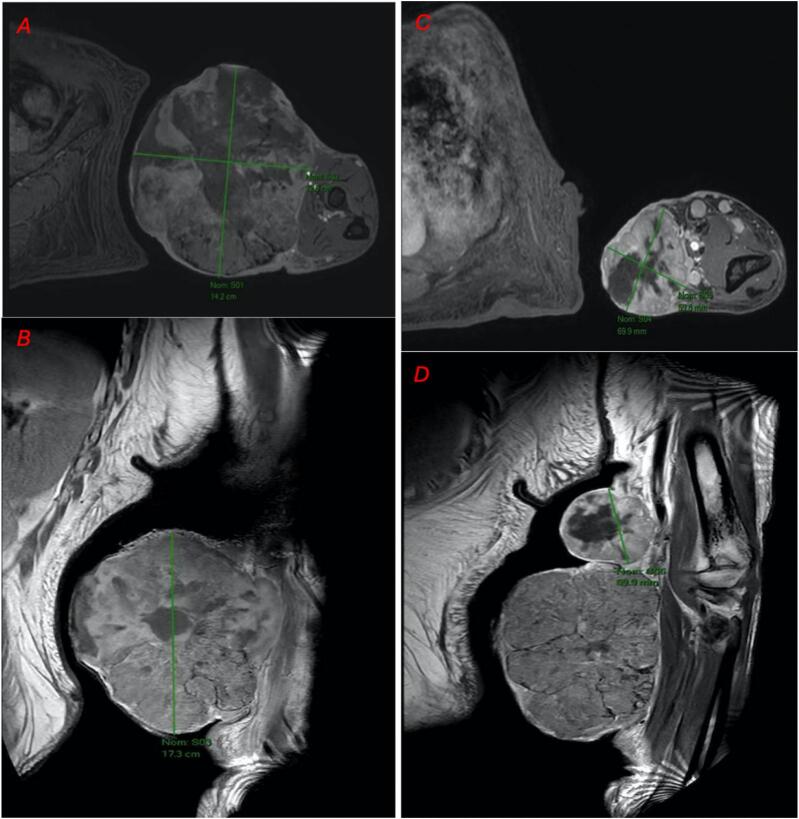
Magnetic Resonance Imaging revealed the two large masses on the left forearm. First large mass measuring 16 × 13 cm in T1 ponderation (A) and in T2 ponderation (B). Second mass measuring 7 cm × 8 cm in T1 ponderation (C) and in T2 ponderation (D).

The CT-scan (computer tomography) and the MRI (Magnetic Resonance Imaging) showed the presence of two large, well-vascularized masses without bone or muscle involvement or metastases (Fig. 2). The appearance primarily suggested a sarcomatous origin.

The biopsy revealed a malignant tumor composed of cells showing nuclear pleomorphism, poorly defined cytoplasm, and hyperchromatic nuclei, with numerous mitoses.

Immunohistochemistry demonstrated positive staining for SOX10, PS100, Melan-A, HMB-45, and a diffuse and intense staining for PRAME.

No staining was demonstrated for pancytokeratine, desmine, myogenine, CD45, CD34, CD31 or ERG. Proliferation index estimated by Ki67 was evaluated at 20 %.

These findings show no sufficient argument for a sarcoma and are on the contrary in favor to a malignant melanoma diagnosis.

**Fig. 3:**
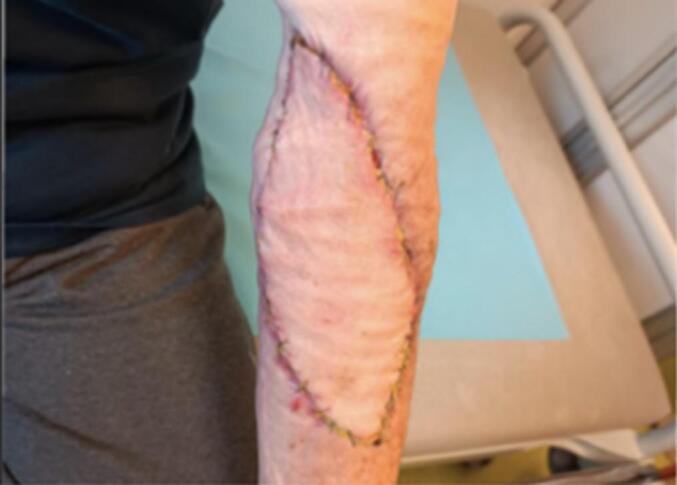
Appearance of the left arm after excision surgery.

Following extended investigation by RNAseq looking for EWSR1 transcripts in order to eliminate any doubts considering a potential clear cell sarcoma diagnosis, which were negative, a multidisciplinary team meeting and negative BRAF testing, a two-stage surgical approach was undertaken: complete excision of the tumor mass with clear surgical margins (10 mm) and subsequent skin grafting (Fig. 3). Excision surgery was possible considering that this melanoma was superficial and did not extend beyond the dermis. It was performed at the beginning of Januray, 2024 by an experienced cervico-facial surgeon, specialized in the management of head & neck and skin cancers.

**Fig. 4:**
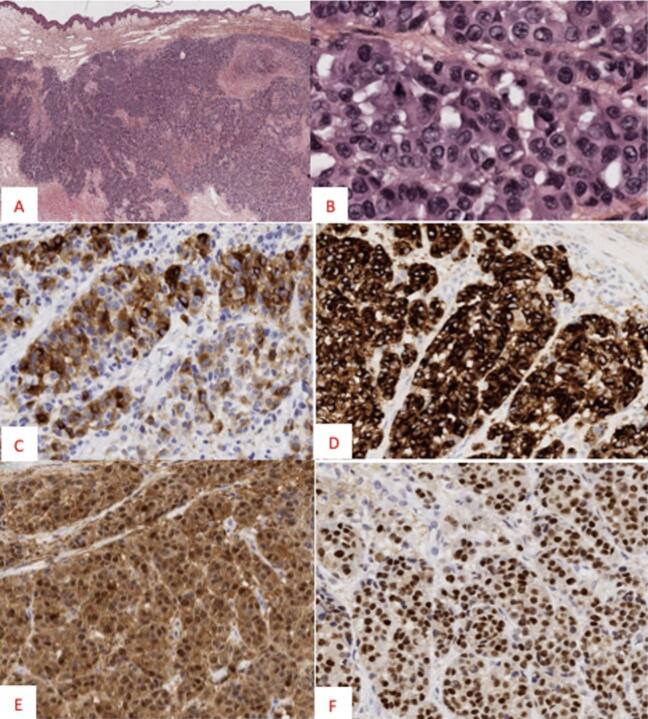
Histological appearance of the forearm tumor. HES stains at ×25 (A) HES stains at ×400 (B) Magnifications showing a nodular tumor made of round cells with solid and nested architectures. Tumor cell expression of MelanA (C), HMB45 (D), PS100 (E) and PRAME (F).

Axillary lymph node dissection was also performed due to the presence of metastatic lymphadenopathy extending to the upper part of Berg level III. Histological analysis of the excised melanoma revealed 80 % necrosis (Fig. 4) with no lymph node metastases; the melanoma was staged as T4N0M0. The tumor showed signs of bacterial superinfection.

Surgery to remove melanoma in the forearm is rarely followed by postoperative complications [2]. In this case, postoperative complications were observed. Significant digestive bleeding occurred, attributed to diverticulosis with diverticular ulceration confirmed via colonoscopy.

**Fig. 5:**
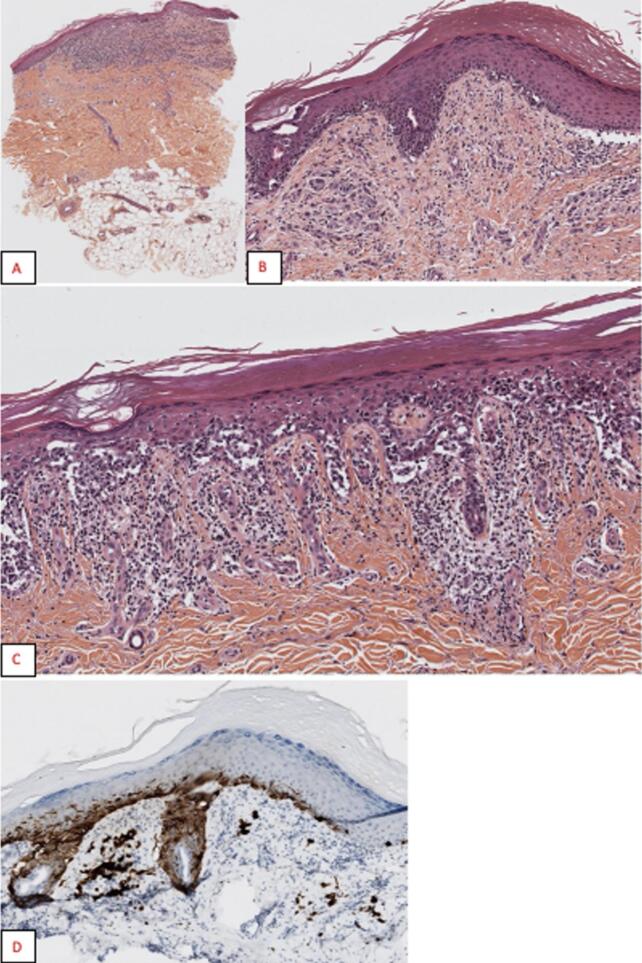
Histological appearance of the right internal malleolus. HES stain of the biopsy (A) showing a superficial lesion; HES stains of the surgical specimen at ×50 (B) and ×100 (C) magnifications, respectively showing an invasive tumor made of round cells with small nested architecture (B) and a lateral lentiginous in situ component (C) causing clefting artifacts. In immunohistochemistry, tumor cells express MelanA (D).

In January and February 2024, the patient presented with new lesions: a nodular basal cell carcinoma on the right cervical region and an acrolentiginous melanoma (Breslow index 0.25 mm, Clark level II with in situ lateral component – Fig. 4) on the medial malleolus of the right foot (Fig. 5). Both were surgically excised with clear margins.

The patient has been receiving immunotherapy with Pembrolizumab since March 2024.

## 3 Discussion

In France, melanomas are increasing in frequency. The global incidence has also increased, particularly in older age groups and in males [3]. The average age of diagnosis is around 60 years old, and the main risk factor is exposure to the sun [4]. There are other risk factors such as family history of skin cancer and male gender. Treatment strategies vary depending on the TNM staging, lesion thickness, ulceration, and extent of disease, with surgery often being the primary treatment modality. Survival after development of a melanoma and medical care depends on these prognosis factors and presence of metastasis. Surgery is the standard treatment [5].

This case illustrates the importance of specialized management of melanomas and extended immunohistochemistry analysis, which allows accurate diagnosis, leading to adapted management of the disease. Indeed, melanomas are sometimes hard to diagnose: in a study published in 2016 by Sondermann, 30 out of 107 patients with acral melanoma were misdiagnosed [6].

Patients who develop melanoma are at higher risk of presenting new skin lesions. Our patient has developed multiple skin tumors after his first bulky melanoma, which asks the question of a possible link between the lesions. In this case, the left forearm lesion did not extend beyond the dermis and there was no metastasis. It is most likely that the lesions developed independently.

Concerning patient's history, a type II diabete was diagnosed during the ER visit. During the following consultations, the patient mentioned an important psychological suffering. He lost his wife 20 years ago and had recently been taking care of a new partner showing signs of dementia, which, he said, greatly affected him. The obvious way this psychological suffering affected his health relies in the observation that the patient was no longer maintaining his personal well-being. He didn't seek medical advice for his arm mass, while it was growing for months and painful. On another aspect, it is now known fact that there may be a link between depressive symptoms and cancer. Accumulating evidence suggests that depressive symptoms, both affective (emotional) and somatic (physical), frequently precede a cancer diagnosis [7,8]. Studies have documented affective symptoms appearing several months before diagnosis, accompanied by somatic manifestations such as fatigue, anorexia, and insomnia [9]. These somatic symptoms are extremely common among cancer populations and may reflect the disease itself, comorbid depression, or both [10]. The onset of these symptoms, sometimes beginning up to 2–7 months before diagnosis [11], suggests a shared underlying mechanism potentially impacting tumorigenesis. This pre-diagnostic manifestation is significant, as depressive symptoms have been reported in up to 30 % of women before a breast cancer diagnosis [12], highlighting a potential causal link.

Moreover, chronic stress significantly elevates the risk of chronic diseases by amplifying pro-inflammatory signals through increased cytokine production and reduced sensitivity to hormonal control via the hypothalamic– pituitary–adrenal (HPA) axis [13]. This pro-inflammatory milieu fosters oxidative and nitrosative stress, potentially leading to mitochondrial dysfunction and DNA damage [13]. A substantial body of preclinical and clinical evidence confirms inflammation's essential role in tumor growth and progression [14]. Pro-inflammatory cytokines, released by tumor and stromal cells, as well as tumor-associated macrophages and infiltrating T cells, drive tumor angiogenesis, metastasis, and potentially contribute to therapy resistance [7,8]. Stress also enhances angiogenesis via increased adrenergic transmission, stimulating cytokine production (e.g., IL-6, IL-8, TNF-α) and VEGF production [15].

The hypothesis that the patient presented an inflammatory microenvironment can be formulated and sustained by the bacterial infection that was observed. Indeed, bacterial infection implies ensuing inflammation responses [16]. Bacteria can cause DNA damage in host cells [17] and disrupt cellular signaling pathways that regulate cell proliferation, apoptosis, differentiation, and immune responses [16], thereby modifying the immune landscape in tumor microenvironment.

This case highlights the challenge in differencing bulky sarcomas from melanomas. Differential diagnosis between clear cell sarcoma and melanoma could be difficult depending on cases, and their management is different [18]. Classification and diagnosis of melanomas is sometimes questioned by pathologists which emphasizes the need for standardized procedures and histological marking [19,20]. Histological markers used to diagnose melanoma are: S100 protein, HMB45, Melan-A, Tyrosinase, MITF and Vimentin [21]. These markers are not 100 % sensitive, there are reported cases in the literature of sarcoma expressing some of these markers [22]. PRAME (PReferentially expressed Antigen in MElanoma) is a frequently positive marker to distinguish melanoma from other cancers. In a study published in 2018 by Lezcano C., carried out on 400 melanocytic tumors, 87 % of metastatic melanomas and 83.2 % of primary melanomas were positive for PRAME [23]. In another study carried out in 2020 on 155 cases of metastatic melanoma, 97.4 % of melanomas expressed PRAME diffusely. Therefore, despite the immunohistochemical similarities between malignant melanomas and sarcomas, PRAME remains a good diagnostic marker for melanomas [24].

Sarcoma histological markers are sometimes common with melanomas detection (for example, S100 protein, vimentin, exceptionally HMB-45 and Melan-A [22]). Liposarcomas express specifically HMGA2, MDM2 and myxoid liposarcomas express specifically CD34 and DDIT13. CD4 and desmine are markers expressed exclusively by liposarcomas and help differentiate them from melanomas.

In cases published in the literature, neoadjuvant treatment with immunotherapy or chemotherapy was necessary for the management of bulky melanomas [22,25,26]. Our excisional surgery for the management of his extensive melanoma demonstrated that although the procedure initially appeared complex, it facilitated a favorable recovery outcome for the patient.

## 4 Conclusion

Large melanomas, although rare, present significant clinical challenges. This case demonstrates the potential for an initial misdiagnosis, which was avoided using immunohistochemistry, allowing for appropriate therapeutic management. The excision surgery played a significant role in patient's recovery, which shows that, when possible, surgery allows favorable recovery outcome in bulky melanoma management. This case also emphasizes the potential impact of psychological stress, bacterial infection and the induced inflamed environment in tumor progression.

## Author contribution

Emma Quack: wrote the paper, collected data regarding the patient, contributed to the bibliography.

Hicham Fatouaki: wrote an initial version of the paper, retracing patient's history, anatomopathological results and collected images (histology, surgery, post-surgery images).

Emma Afri: participated in writing the paper and collecting data, contributed to the bibliography.

Dr. Romina Mastronicola: provided her expertise and advice on this case, discussed it with Pr. Gilles Dolivet, read and corrected the paper.

Pr. Gilles Dolivet: Performed the surgery, conducted the study, designed the study, collected data, interpreted data, participated in writing the paper, discussed it with Dr. Romina Mastronicola.

## Consent

Written informed consent was obtained from the patient for publication and any accompanying images. A copy of the written consent is available for review by the Editor-in-Chief of this journal on request.

## Ethical approval

Ethics Committee of the Lorraine Cancer Institute. There is no reference number as all patients give written consent by signing a consent form at their first appointment.

## Guarantor

Emma Quack.

## Research registration number

Not applicable.

## Funding

This research did not receive any specific grant from funding agencies in the public, commercial or not-for-profit sectors.

## Methods

All work has been reported in line with the SCARE criteria [27].

## Conflict of interest statement

The authors declare no conflicts of interest.
